# Microplastics in aquatic systems, a comprehensive review: origination, accumulation, impact, and removal technologies

**DOI:** 10.1039/d2ra04713f

**Published:** 2022-10-04

**Authors:** Antonio Tursi, Mariafrancesca Baratta, Thomas Easton, Efthalia Chatzisymeon, Francesco Chidichimo, Michele De Biase, Giovanni De Filpo

**Affiliations:** Department of Chemistry and Chemical Technologies, University of Calabria Via P. Bucci, Cubo 15D, 87036 Arcavacata di Rende (CS) Italy antonio.tursi@unical.it; School of Engineering, Institute for Infrastructure and Environment, University of Edinburgh The King's Buildings Edinburgh EH9 3JL UK; Department of Environmental Engineering, University of Calabria Via P. Bucci, Cubo 42B, 87036 Arcavacata di Rende (CS) Italy

## Abstract

Although the discovery of plastic in the last century has brought enormous benefits to daily activities, it must be said that its use produces countless environmental problems that are difficult to solve. The indiscriminate use and the increase in industrial production of cleaning, cosmetic, packaging, fertilizer, automotive, construction and pharmaceutical products have introduced tons of plastics and microplastics into the environment. The latter are of greatest concern due to their size and their omnipresence in the various environmental sectors. Today, they represent a contaminant of increasing ecotoxicological interest especially in aquatic environments due to their high stability and diffusion. In this regard, this critical review aims to describe the different sources of microplastics, emphasizing their effects in aquatic ecosystems and the danger to the health of living beings, while examining, at the same time, those few modelling studies conducted to estimate the future impact of plastic towards the marine ecosystem. Furthermore, this review summarizes the latest scientific advances related to removal techniques, evaluating their advantages and disadvantages. The final purpose is to highlight the great environmental problem that we are going to face in the coming decades, and the need to develop appropriate strategies to invert the current scenario as well as better performing removal techniques to minimize the environmental impacts of microplastics.

## Abstract 2

Although the discovery of plastic in the last century has brought enormous benefits to daily activities, it must be said that its use produces countless environmental problems that are difficult to solve.

## Graphical abstract

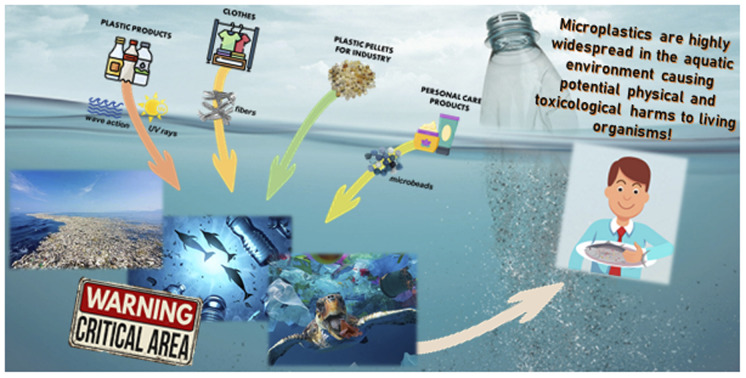

## Introduction

Growing environmental alarm has arisen recently due to the presence of plastic waste in aquatic systems. The generation of anthropogenic waste, 70% of which is plastic, has increased exponentially in the last decades.^1^ In fact, more than half of plastic becomes waste in less than a year from production and most of it is not recycled or reused. Microplastics are found all over the world, from the poles to the equator, from coastal regions to aquatic ecosystems. Their diffusion is massive due to transport phenomena such as wind and ocean currents which also lead to their presence in other ecosystems.

**Fig. 1:**
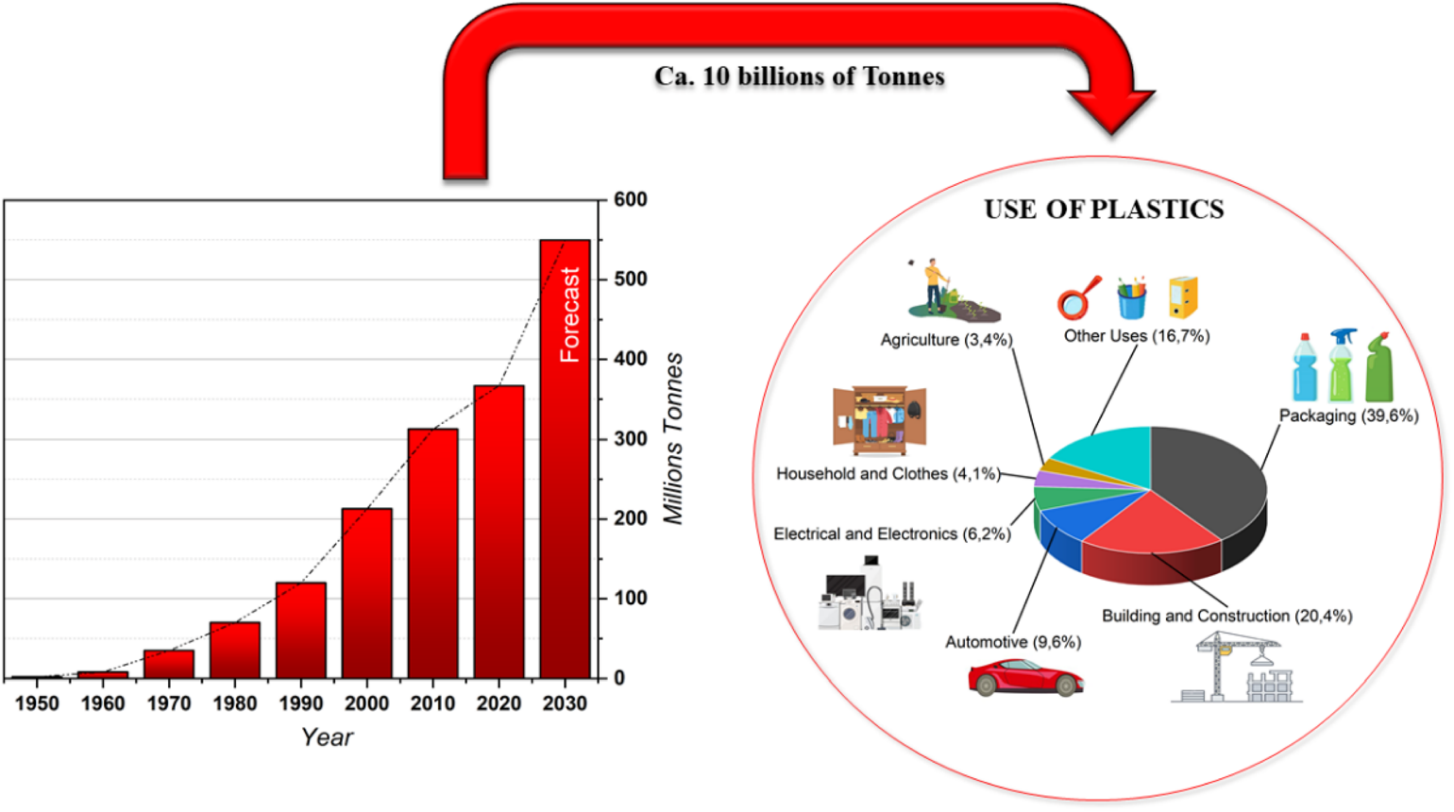
Annual production of plastics worldwide from 1950 to 2020 (in million metric tons) and their use.^2^

Since the 3rd industrial revolution in 1950 more than 10 billion tons of plastic have been produced with the annual production rate increasing exponentially. To be more specific, plastic production massively increased from 2 million tons in 1950 to 367 million tons in 2020 (about 0.3 percent less than in 2019 due to the impacts of COVID-19 on the sector).^2^ Furthermore, it is estimated that production will further increase to about 600 million tons in 2025 (Fig. 1).^2^

The incredible versatility of plastic materials explains the continuous growth of production year after year as well as their market value.

Of all the plastic produced, recent studies have shown that only 9–10% has been recycled, another 10–11% was incinerated and *ca.* 30% is still in use due to their long lifetime. The remaining 50% has been disposed of in landfills or dispersed into the environment.^2^

One of the biggest problems, in this case, is precisely the fact that much of the plastic dispersed in the environment can easily reach the rivers and oceans. In fact, according to the latest 2020 survey, plastic waste enters the ocean at a rate of approximately 11 million tons per year.^3^

Plastic pollution is particularly acute in estuaries, indicating that terrestrial river input is the preferential way of pollution in coastal and marine environments.^4,5^

Furthermore, the COVID-19 pandemic has triggered an estimated global use of 129 billion masks and 65 billion gloves every month, generating a further release of plastics into the environment and therefore into the oceans.

Recent studies provide some truly dramatic evidences; 5.25 trillion macro and micro pieces of plastic float in our ocean with 46 000 pieces in each square mile, weighing up to 269 000 tons.^3^ Macroplastics (diameter ≥ 5 mm) are a problem affecting the marine environment both from an aesthetic and environmental point of view with enormous repercussions on the marine biota. For example, plastic ingestion and entanglement in debris are the main cause of injury and death to mammals, fish, reptiles and seabirds.^1,6^

Moreover, microplastics (MP) with diameter < 5 mm can have detrimental impacts to organisms, including humans, since, due to their small size, they bioaccumulate in organisms throughout the food chain. In addition to this, MP can be of greater concern than macroplastics because, due to their high surface area and their distinctly hydrophobic character, they tend to absorb many pollutants such as heavy metals,^7,8^ polycyclic aromatic hydrocarbons (PAHs) and polychlorinated biphenyls (PCBs),^9,10^ and to transfer them to marine fauna, thus entering the food chain.^11,12^ Carpenter and Smith (1972) have been the first to highlight the presence of small plastic fragments in the open sea.^13^ Plastic waste can also strongly affect the ecosystem by generating new habitats on floating plastic debris,^14^ opacifying the seabed and creating a barrier that interposes between the sea surface and the atmosphere with consequent limitation of gas exchange between the two ecosystems.^6,7^ Nowadays, the largest reports of large quantities of plastic waste come mainly from areas located in subtropical latitudes, where concentrations of plastic waste, carried by currents and winds, accumulate on the surface of the sea, forming real oceanic islands called “garbage patches”. Mass concentrations per km^2^ reach hundreds of kilograms, counting up to one million pieces, for particles with a size > 500 μm. In light of these facts, the pervasiveness of MP waste in aquatic ecosystems as a result of anthropogenic pollution has received scientific attention worldwide. The methods currently being studied for their removal include absorption, filtration, biological degradation and/or chemical treatment processes.

Alongside the aforementioned methods, in recent decades efficient techniques for removing MP from wastewater, a main source of MP discharge to receiving water bodies, have been eagerly required to increase the quality of the final effluents and mitigate MP pollution. Several advanced treatment technologies have been studied through the use of membrane bioreactors for the treatment of the primary effluent and various tertiary treatment technologies such as disc filters, rapid sand filtration and dissolved air flotation for the purification of the secondary effluent.^15,16^ However, there is still ample room for improvement and optimization of such ad-hoc technologies until wastewater treatment legislation enforces their application in existing wastewater treatment plants (WWTPs). Furthermore, many recent studies are focusing on the filter systems themselves, investigating the possibility of using natural polymers and eco-friendly materials to replace synthetic ones, in order to reach both comparable remediation efficiencies against several pollutant and plastic waste reduction at the end of their life cycle.^17–21^ This review presents recent advances in understanding the impacts of MP on the environment and humans as well as the current state of the art on developing appropriate removal technologies. For this purpose, the major sources of MP pollution and their classification are reported. Recent studies focusing on environmental and human health impacts are reviewed. Furthermore, physical, chemical and biological technologies for MP removal from wastewater are assessed, also considering the latest advances in the scientific field to identify the gaps in the sector and guide future research priorities.

## Classification and sources

### Plastic classifications

**Fig. 2:**
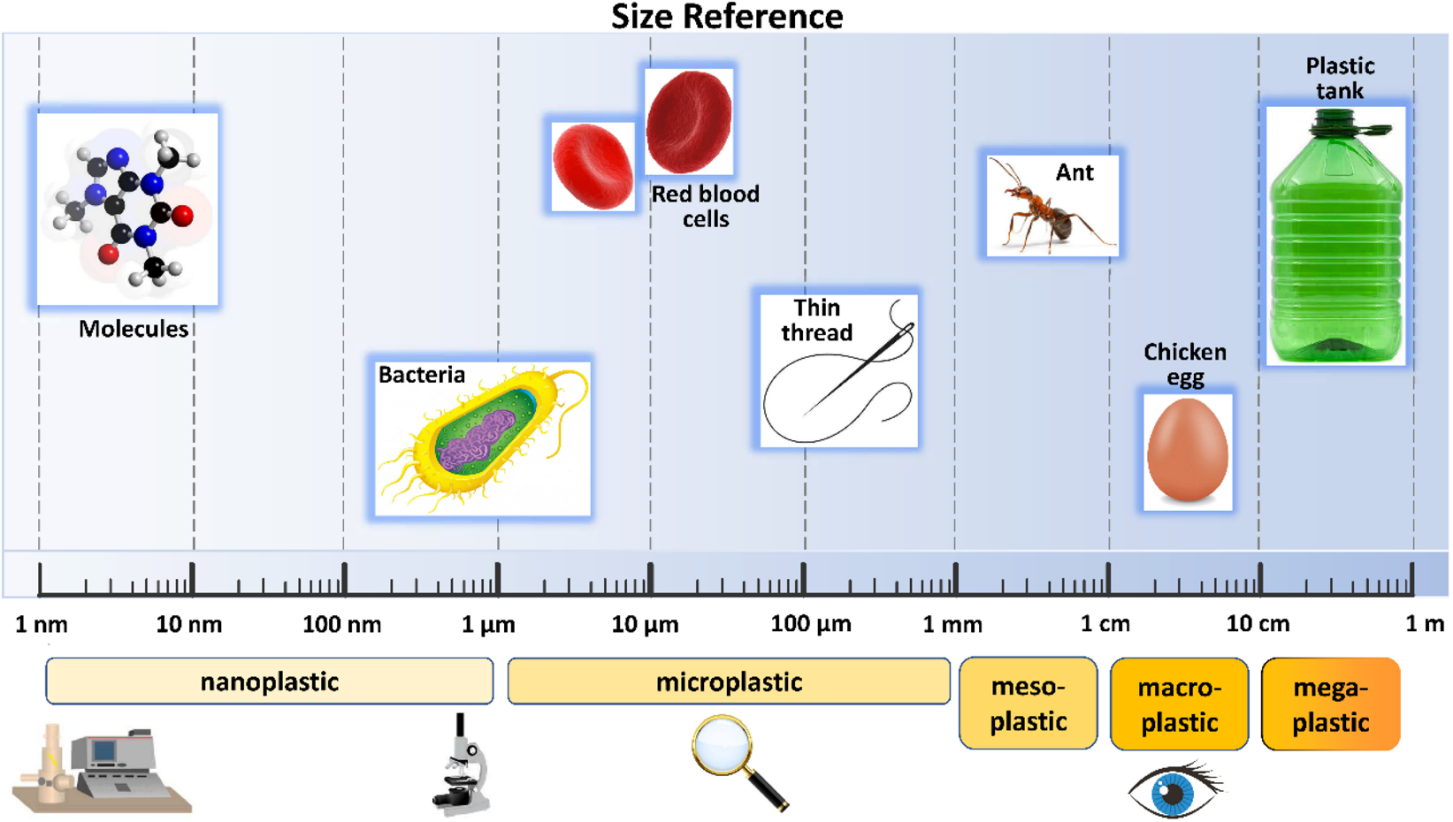
Size classification of plastic particles.

In aquatic systems, plastic particles differ in shape, size, chemical composition and specific density.^22^ Currently, the most widely adopted classification is based on their size (Fig. 2). According to this, plastic debris is divided into four categories: megaplastic (>50 cm), macroplastic (5–50 cm), mesoplastic (0.5–5 cm) and microplastic (<0.5 cm).^23^ In 2011, Andrady^24^ introduced the concept of nanoplastics, defining them as particles with sizes between 200 nm and 2 μm. A few years later, in 2015, Jambeck *et al.*^25^ set the upper size limit of nanoplastics at 100 nm. A brief description of the most common characteristics of the above mentioned plastic is provided below.

Megaplastic (MegP) and macroplastic (MaP) are characterized by large debris, visible to the naked eye. Although they are considered one of the major source of marine plastic pollution, they have garnered increasing attention from the scientific and social community only in recent years.^26,27^ Greater amounts of MegP and MaP are released from disposable products, being produced in large quantities and used for a relatively short time.^28^ Winton *et al.* (2020), in fact, showed that bottles and lids (7.51% of all litter), bags (5.49%), food wrappers (8.92%), cigarette butts (4.02%), smoking-related packaging (3.40%) and sanitary items (3.72%) are commonly plastic products found on European beaches.^29^ The main risk induced by mega/macroplastic for living organisms is represented by the possibility of entanglement and ingestion, which may cause their immediate death by suffocation. This is particularly evident for fish, marine mammals and birds, living in the environments currently most polluted by plastic.^27^

Mesoplastic (MesP), mainly deriving from degradation of macro and megaplastic, exhibits particle sizes between these two classifications. Currently, the number of articles investigating the presence and characteristics of mesoplastic in the marine environment is increasingly growing. Recent studies have shown that MesP density, measured in terms of items per m^2^, increases considerably during summer seasons in the most South-American touristic sites.^30^ Blettler *et al.* (2017) observed that foam plastics are the dominant mesoplastic category, characterized by many different colors and therefore giving evidence of a high variation in polymer type and origin source.^31^ However, the number of MesP particles dispersed in the environment is always smaller than that of MP.^32,33^ Isobe *et al.* (2015) found that the concentration of mesoplastic is about 10 times lower than that of MP in East-Asian seas, with a number of collected debris of about 12 000 per MP compared to 780 of MesP and an average concentration of 3.74 and 0.38 pieces per m^3^, respectively.^34^

**Fig. 3:**
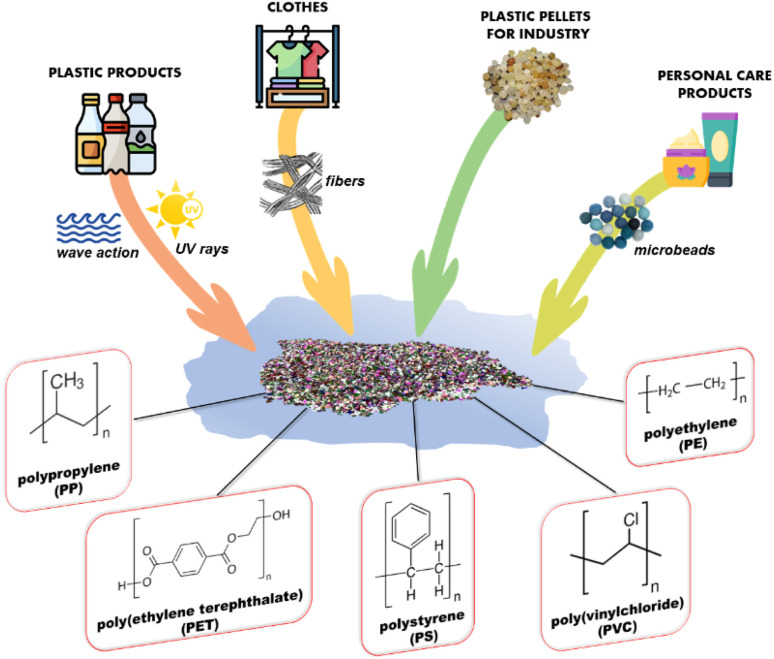
The main sources of MP pollution and the most common plastic polymers present in the environment.

Plastics at different sizes can be generated by fragmentation and degradation of debris^35^ due to physical forces, such as waves and currents in aquatic systems, and to environmental and atmospheric conditions, such as solar radiation, pH and temperature. Physical and chemical characteristics of plastics also play a major role in the fragmentation and degradation processes. The most common plastic polymers present in the environment are polypropylene (PP), poly(ethylene terephthalate) (PET), polyethylene (PE), polystyrene (PS), and poly(vinylchloride) (PVC) (Fig. 3). Their chemical and physical properties, sources, and average quantities produced annually have been reported in Table 1.^24^

**Table 1:** Chemical–physical properties, sources, and average quantities produced annually (2020) of the common microplastic wastes

Plastic class		Specific gravity	Percentage production	Products and typical origin
Polypropylene	PP	0.83–0.85	19.7	Food packaging and wrappers, caps, microwave containers, pipes, automotive parts, *etc.*
Low-density polyethylene	PE-LD	0.91–0.93	17.4	Reusable bags and containers, agricultural film, food packaging film, *etc.*
High-density polyethylene	PE-HD	0.94	12.9	Milk bottles, toys, shampoo bottles, houseware, *etc.*
Poly(vinyl chloride)	PVC	1.38	9.6	Window frames, profiles, floor and wall covering, pipes, cable insulation, garden hoses, *etc.*
Polyethylene terephthalate	PET	1.37	8.4	Bottles for water, soft drinks, juices, cleaners, *etc.*
Polyurethane	PUR	1.05–1.28	7.8	Building insulation, pillows and mattresses, insulating foams for fridges, *etc.*
Polystyrene	PS	1.05	6.1	Food packaging, building insulation, electrical and electronic equipment, eyeglasses frames, *etc.*
Other plastics	—	—	7.4	Phenolic resins, epoxide resins, melamine resins, urea resins, *etc.*
Other thermoplastics	—	—	10.7	Hub caps (ABS); optical fibres (PBT); eyeglasses lenses, roofing sheets (PC); touch screens (PMMA); cable coating in telecommunications (PTFE); aerospace components, medical implants, surgical devices, membranes, protective coatings, *etc.*

### Pollution sources

**Fig. 4:**
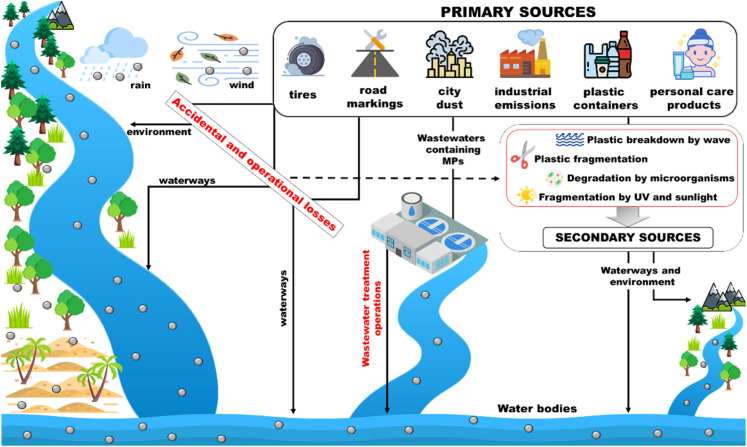
Primary and secondary sources of MP pollution.

Microplastics pollution sources can be divided into two main groups, namely primary and secondary, based on their origin. The distinction is based on whether the particles were originally produced with those dimensions (primary) or whether they derived from the degradation and/or breakdown processes of larger debris (secondary) (Fig. 4).

#### Primary sources

The main sources of primary MP are tires, road markings, marine coatings, synthetic textiles, personal care products, plastic pellets, city dust,^36–39^ which flow into the environment mainly through domestic sewage, WWTPs or atmospheric events.^40,41^ In particular, transporting tires and road wear particles (TRWP) are dispersed through rainwater as a transport route,^42,43^ synthetic fibers deriving from clothing, personal care products like scrubbers in cosmetics, artificial grass, landfills and waste incineration^38,44^ are carried by the wind in the aquatic environment or deposited in the terrestrial environment. While large plastic particles can be efficiently removed during wastewater treatment, MP often bypass treatment units, entering and accumulating in the aquatic environment.^45^ In fact, most WWTPs are located close to water courses, seas or oceans, thus inducing a more copious and simplified release of microplastics due to the shorter path. For example, in mainland China, more than 50% of wastewater treatment plants (1873 out of 3340), with a treatment capacity of around 10^7^ m^3^ day^−1^, are located in coastal regions where their effluents can discharge directly into aquatic ecosystems.^46–48^ Recent studies have highlighted that agriculture is also one of the main anthropogenic activities that generates MP pollution mainly in the soil due to the use of sewage sludge as soil improvers and the use of agricultural plastics, such as plastic mulches.^49,50^ However, soil MP pollution can be easily transferred to aquatic systems through agricultural runoffs. MP release can also occur at various stages of the life cycle of plastic products (*e.g.* manufacturing, transportation or recycling). Nevertheless, most leaks occur mainly during the use phase of plastic-containing products, which is the main reason that several countries have banned the use of MP in personal care products.^51^ In addition, the European Union has called for the use of specially added microplastics (for example in cosmetics and personal care and cleaning products) to be banned by 2020. They have also proposed stricter rules to significantly reduce the unintentional release of microplastics from products such as synthetic fabrics, tires, paints and cigarette butts. The strategy adopted by the EU also includes the use of single-use plastics. In December 2018, European legislators, Parliament and Council, approved a ban on the use of certain single-use plastic products, such as cutlery, plates and balloon sticks, and the obligation for manufacturers of plastic packaging to contribute to the costs of waste collection for these products.

#### Secondary sources

Secondary MP are defined as micro-waste resulting from the breaking up of larger plastic debris through physical, biological and chemical degradation processes. These processes are mainly (a) photodegradation by sunlight (mainly caused by exposure to UV-B radiation); (b) mechanical degradation such as wave action and sand friction;^52^ (c) thermo-oxidative degradation or oxidative erosion; (d) biodegradation by microorganisms that can degrade the hydrocarbons of plastics;^53^ and (e) hydrolysis by sea water. Each of these processes can take place individually or simultaneously on a plastic fragment, depending on the ambient environmental conditions. Degradation is usually increased on beaches and offshore, where plastic debris is subjected to more extreme conditions than those found in continental areas or in inland water bodies. All processes can lead to a considerable decrease in the average molecular weight of the polymer and therefore to a drastic reduction of its dimensions, generating secondary MP. In the presence of high oxygen concentrations (*i.e.* in winter and early spring, when the water temperature is low, the dissolved oxygen concentration is high^54^) the degradation processes, especially those of photodegradation or bacterial biodegradation, are much more favoured. In some cases, polymers can self-catalyze the degradation processes leading to the generation of oxygen-rich substances. Such fragmentation and degradation processes increase the availability of plastic debris in the environment, posing additional environmental risks.^55^

Although the degradation processes that can occur in aquatic and coastal environments are different, their bio-decomposition rates are not sufficiently fast to cause a beneficial effect towards environmental dispersed plastics.

The slow degradation is mainly due to the temperature, the pH of the sea water but above all to the scarce presence of microbial species capable of degrading these polymers. Furthermore, fouling-defouling, meaning the accumulation of encrustations on the surface of floating plastics, and their subsequent sinking, alternated by the enrichment of foraging bacteria that make the detritus regain buoyancy, can generate a continuous change of environmental conditions along the water column, such as to negatively interfere with the degradation process. Zbyszewski *et al.* (2014)^52^ highlighted how studying the degree of surface aging of microplastics can be useful for tracing the history of particles. Furthermore, the evaluation of plastic degradation patterns in different ecosystems is fundamental to understand how particles interact with the environment and how various factors influence their stability, transport and final fate.^56^

## Environmental and accumulation cycle of microplastics

### Fate and accumulation

**Fig. 5:**
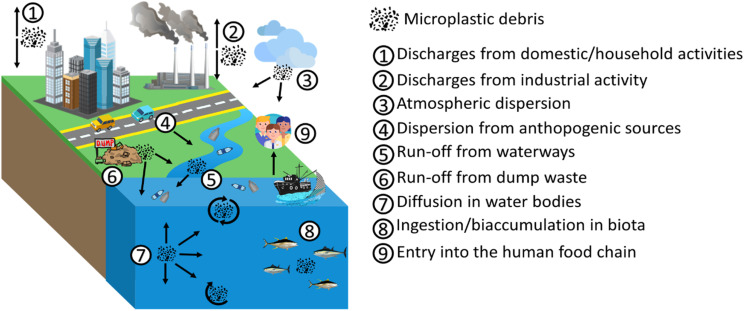
Pathway example of MP diffusion.

**Fig. 6:**
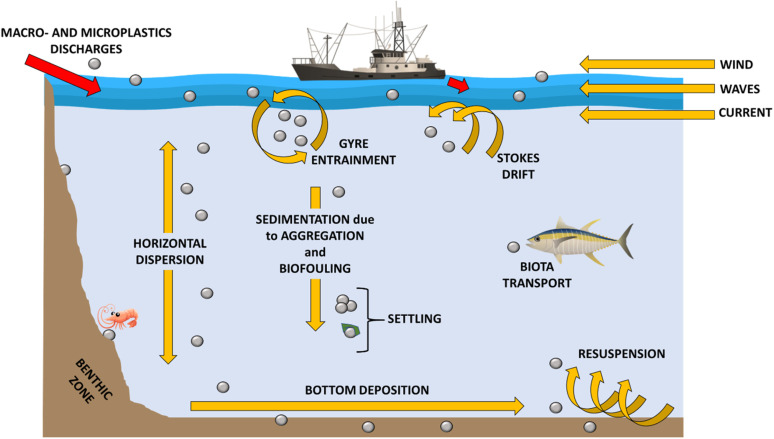
Microplastic diffusion in the aquatic system.

The spread and cycle of microplastics from urban and industrial settlements to rivers and lakes, as well as transport to the sea and subsequent marine dispersion on the surface and deep in ocean basins have been intensively studied^57–60^ (Fig. 5). Plastic waste is usually generated (a) by the inhabitants, varying according to their habits, geographic location and existing infrastructures; (b) from waste management and treatment, also including collection and transport; (c) from industrial and manufacturing plants (Fig. 5). Plastic waste can take different paths spanning from reuse to recycling, to incineration, to landfill disposal and to dispersion into the environment. The most environmentally friendly practices are reuse or recycling, which keep the plastic in a closed loop (*i.e.* the material remains in the value chain). The local distribution of MP is strictly dependent on the complex interactions between the sources of plastics, their dispersion and the current environmental conditions. Therefore, the distribution and fate of microplastics is highly heterogeneous and challenging to monitor.^24^ Despite this, predictive models of MP diffusion have been developed over the last few years. Diffusion processes can be substantially influenced by the geography of the territory, by physical, chemical and biological processes, mainly related to atmospheric conditions, and by the physical properties of the fragments (*e.g.* size, shape, density, buoyancy).^61–63^ It has been found that most MP particles released to land will finally end up in the marine environment.^25,58,64,65^ Especially in coastal regions, terrestrial sources are considered an important contribution to marine plastic debris,^25^ with between 1.15–2.41 million tons per year of plastic waste being carried into the ocean by rivers.^64^ Schmidt *et al.* (2017)^65^ also revealed that rivers are a preferred route contributing between 80% and 94% of the total plastic load entering the seas and oceans. Moreover, a strong correlation between population density and microplastic concentration has been verified. The presence of densely populated regions and inappropriate waste management can generate high levels of contamination.^66–68^ From the evidence set out so far, the global distribution of MP in the marine environment is well established. Their ubiquitous presence in the marine ecosystem leads to high interaction with the biota both in surface waters and in the deepest abysses, as well as in sediments.^69^ Over 1400 marine species interact, primarily through entanglement and ingestion, with marine plastic debris in different ways.^70^ MP are usually mistaken for food due to their micrometric size and variable coloration. A wide variety of marine biota such as corals, zooplankton, phytoplankton, lobsters, sea urchins and fish ingest microplastics and, based on their movements, transfer them to remote or pristine areas.^71^ Larger marine biota creatures such as sea turtles, whales, sharks, polar bears and seals are also susceptible to ingesting MP in the oceans.^72^ In addition to direct ingestion from water, MP can be ingested from marine biota through their prey.^73,74^ In particular, the species that feed on phytoplankton can ingest MP following the formation of the aggregates that the latter generate with photosynthesizing autotrophic organisms. As for the predatory vertebrate species, they can involuntarily ingest MP, confusing the synthetic microparticles for the prey, but also by ingesting invertebrates containing MP (*i.e.* bivalves, amphipods, barnacles, polychaetes) favouring their trophic transfer. Therefore, the variable pathways of MP suggest that all marine organisms, from those inhabiting the abyssal depths to those occupying surface waters and benthic zones, are exposed to MP contamination. This transfer of species to species and the different interactions in the various food compartments ultimately generate channelling towards the human food chain. Microplastics suspended in the ocean water column or dispersed on the sea surface can be transported from their release zone to remote areas^75^ (Fig. 6).

However, most of these micro-fragments can accumulate in the central oceanic regions (termed “gyres”).^76^ An oceanic gyre is a system of ocean currents that move in a circular pattern. They are created by variations in the winds direction and forces generated by the rotation of the Earth. The oceanic gyre is not fixed on a particular point in the ocean but moves to coincide with the wind patterns. These patterns are known to drive the “ocean conveyor belt” that circulates ocean water across the planet. The most notable oceanic gyres include the Indian Ocean Gyre, the North Atlantic Gyre, the South Atlantic Gyre, the North Pacific Gyre, and the South Pacific Gyre. These ocean current systems cause the movement of plastic debris but, at the same time, lead to their accumulation. Cozar *et al.* (2014)^76^ estimated that the result of ocean gyres led the Pacific Ocean to contain about 35% of the global amount of ocean plastic.^77^ Therefore, non-entrained plastics and MP in gyres can reach remote ocean regions and coasts as a result of ocean transport caused by surface currents, bottom water transport, thermohaline circulation, Coriolis force and friction with air currents.^77^

A further factor linking the MP distribution and the action of the wind is the so-called “Stokes drift”, as well as a combination of surface residual currents. These phenomena generate the transport of MP from the open sea to shallow coastal waters.^78,79^ The evaluation of the different factors influencing the distribution of microplastics in marine region has allowed to develop models to quantify the expected plastic load in a given region as a function of time, in order to locate areas of high accumulation of debris and potential threats.^76,80^

Many scientists have applied transport models of waterborne materials and particles (oil spills, larvae, sediment) to study the Lagrangian trajectories of surface microplastics.^63,81,82^

The following are the most used models for this purpose:

(1) NEMO: Lagrangian tracking model, used to predict both the accumulation of floating debris and its stranding points in the Mediterranean area.^83^ This model, based on repeated 1 year predictions with 24 hours evaluations, took into account an initial homogeneous distribution of marine debris;

(2) MEDSLIK-II: prediction model of the movement of plastic debris in the Adriatic Sea, starting from an estimate of 10 000 tons per year of waste released, with an entry point, at increasing volumes of plastic, identified in the northwest region of the Adriatic, and no defined accumulation points. The model highlights that the main accumulation points are represented by the seabed and coastal areas;^78^

(3) PLETS-2D: Lagrangian model of particle tracking, used to predict the trajectories in 90 days of debris from different entry points. The study showed that wind drift greatly influences particle distribution, generating sources of uncertainty in the mapping of plastic distribution;^84^

(4) Pol3DD: Lagrangian simulation model of particle tracking to predict floating debris in the world ocean. This model highlighted the high concentration of plastic debris in subtropical gyres.^85^

Although many of these models have proved effective, they are subject to uncertainty, both because of the changeableness caused by the short-term variation of abiotic factors, such as the direction and strength of the wind, and because the considered factors are affected by sources of uncertainty determined by the global climate change (coral bleaching,^86^ ocean acidification,^87^ and ice melt in polar regions^88^ affecting our oceans).

Indeed, these changes, in addition to influencing the ability of models to predict the position of plastic aggregates, could also alter geographic areas and ecosystems due to the negative effects of marine plastic pollution.^63^

In addition to induced extrinsic turbulence, a key role also derives from the intrinsic characteristics of microplastics such as size, density and shape that modify their speed of adoption.^62,89,90^ Once MP reach the marine ecosystem, their density greatly influences their distribution. Those with a lower density than sea water and a neutral surface charge float to the surface or disperse in the water column, while those with higher density tend to accumulate in benthic environments,^79,89^*i.e.* those ecological zones at the lowest level of a basin or sea, also including the sediment surface and some subsurface layers. Taking into account that more than 65% of the produced plastics have a lower density than sea water, the largest fragments are found floating on the surface or mixed in the surface water column of the oceans.^63,89^ At this point, MP are dispersed by dynamic conditions, such as wind strength and geostrophic circulation, resulting in a very wide variability in surface concentrations.^81,91^ As far as high-density MP are concerned, these are not floating and tend to settle both in coastal sediments and in the seabed.^92,93^ Recently, experimental researches have been conducted to evaluate the sedimentation behavior of high density microplastics (density higher than that of water).^61,94^ For example, Ballent *et al.* (2012)^62^ studied the sedimentation rate of microplastic pellets having different densities between 1.06 and 1.13 g cm^−3^. The results showed that the sedimentation rate (which varies from 20 to 60 mm s^−1^) is directly proportional to the increase in density.

Neutral surface charge MP can be found on the sea surface but also suspended in the water column or lying in the subsoil of deep waters. These considerations emerged following discrepancies found between the expected plastic concentrations and those actually found in surface ocean waters.^76,80^

Recent studies have proved that the highest concentration of microplastics is found in the intermediate part confirming that the microplastics are distributed vertically inside of the water column, mainly due to the turbulent regimes of the waters, generated by the wind and currents.^95^

Enders *et al.* (2015)^96^ examined the vertical dispersion of microplastics as a function of their size. Research results showed that smaller sized microplastics are much more affected by turbulent mixing. While empirical calculations, resulting from the use of the General Ocean Turbulence Model (GOTM), have shown that particle-shaped microplastics have a higher diffusion rate than sheets, which in turn are more affected by turbulent phenomena than fibrous debris.^97^

At the same time, further studies^61^ have shown that the sinking speed of microplastic fragments is influenced more by shape rather than density (particles of different forms of polyamide (1.14 g cm^−3^) sink faster than those of polyvinyl chloride (1.56 g cm^−3^)).

In addition to the characteristics of shape and density, it is also necessary to take into account the phenomenon of biofouling: microorganisms of different nature can rapidly aggregate on the surface of plastic debris and develop biofilms.^24,93,98^

Còzar *et al.* (2014)^76^ and Moret-Ferguson *et al.* (2010)^99^ hypothesized that the phenomenon of biofouling could increase the density of microplastics to such an extent that particles with a density lower than that of seawater (for example, polyethylene or polypropylene) can reach densities that exceed it, leading to a slow sinking of the particles. At the same time, biofouling can reduce the surface hydrophobicity of microplastics, generating a greater tendency for them to sink:^76,100^ plastic particles that would normally float (such as polyethylene and polypropylene) have been found in marine sediments.^101^ As reported by Chubarenko *et al.* (2016),^94^ heavy microplastic particles take on average less than 24 hours to settle through the 250 m water column, while fibrous plastics, such as polyethylene, take about 6–8 months to sink due to biofouling.

Finally, the sinking speed of microplastics can also be increased by the phenomenon of incorporation into organic aggregates.^102,103^ Long *et al.* (2015)^104^ found, based on their experimental evidence, that the sinking rate of polystyrene microspheres (2 μm, density of 1.05 g cm^−3^) embedded in diatom aggregates could reach several hundred meters per day compared to 4 mm per day for freely suspended beads.

## Impact on human health

Microplastics represent a huge problem due to their toxicity and persistence in the environment. Their toxicity is mainly attributed to (a) the presence of hazardous chemical additives during their production process, and (b) to the fact that due to their considerable surface area and hydrophobic character, MP are able to absorb and concentrate many organic and inorganic chemical contaminants (indirect toxicity). It was recently observed that MP can absorb chemical pollutants such as polybrominated diphenyl ethers (PBDEs),^105^ perfluorochemicals (PFCs),^106^ drugs and personal care products (PPCP).^107^ The persistent organic pollutants must also be added to the list, better indicated by the acronym POPs. Among them, MP are particularly prone to absorb polycyclic aromatic hydrocarbons (PAHs), polychlorinated biphenyls (PCBs) and organochlorine pesticides such as dichlorodiphenyl trichloroethane (DDTs). The absorption of these pollutants on MP certainly depends on both the type of polymer and its state (rubbery or glassy). Due to their small size, MP can be easily ingested and/or inhaled by organisms. This therefore implies that, through ingestion and/or inhalation, MP and the environmental pollutants they contain enter the circulatory system of organisms, penetrating into the tissues and organs, which is why toxicological studies are necessary to highlight the real risks deriving from the “assumption” of MP.

### Human exposure to microplastic

Main sources of human exposure to MP are (a) ingestion, of water or food; (b) inhalation, both of indoor and outdoor air; (c) dermal contact, through indoor dust, personal care products and fabrics. Despite their toxicity, risks for human health deriving from exposure to MP are not simple to assess as most of the developed methods provide quantitative data in terms of MP number, size and shape, failing to translate these in terms of dose or mass. A recent study^108^ estimated that the annual amount of MP potentially ingested by a person can fluctuate between 11 845 and 193 200 particles, most of them coming from drinking water consumption.

#### Inhalation

Due to their low density and small size, suspended MP particles can accumulate in the atmosphere and can be easily inhaled by humans. MP concentration in the air can significantly vary depending on the season, the overall air quality,^109^ the characteristics of the particles (density, size, surface charge, hydrophobicity), and the different sampling methods adopted for the analyses. For example, a study in Paris showed that the number of MP in the air significantly increases during the rainy seasons compared to dry ones.^110^ The concentration of MP between indoor and outdoor is also significantly different; generally the values are about 5 to 10 times higher for indoor air.^111^ However, it should be noted that most of the data are expressed in terms of daily number of fibers, particles, fragments or elements per m^2^ (per fallout) or per m^3^ (per air), which can differ significantly from day to day. For example, Dris *et al.* (2017) estimated the indoor MP concentration between 0.4 and 56.5 particles per m^3^ while the outdoor one was between 0.3 and 1.5 particles per m^3^.^112^ Taking into account these data, Prata (2018)^113^ estimated that an adult man can inhale on average, considering a human tidal volume of 6 L min^−1^, a daily number of MP particles between 26 and 130. Furthermore, in 2019 Vianello estimated that, in the case of a completely sedentary life, this daily value can reach up to 272.^114^

The presence of MP particles in the air (both indoor and outdoor) is mainly associated with plastic degradation processes, landfills, synthetic textiles, building materials and waste.

Inhaled MP particles accumulate predominantly in the lungs. The pulmonary alveoli have a tissue barrier of less than 1 micron and a large surface area (150 m^2^) so they constitute an optimal adsorption site for MP. The main risks associated with human health involve the appearance of an inflammatory response of the lung tissue, which may also be followed by cytotoxicity and genotoxicity.^115^ Xu *et al.* (2019) showed a cytotoxic and genotoxic effect on the pulmonary epithelium and macrophages caused by polystyrene particles of 50 nm size.^116^ Lung inflammation can also be chronic, due to the intense release of proinflammatory chemotactic factors, often known as dust overload.^113,117^ Also, it was observed^113^ that workers in textile factories, exposed to acute and chronic inhalation of MP, can easily be subjected to respiratory tract diseases. Prolonged exposure to MP can also lead to lung diseases, including asthma, pneumoconiosis and extrinsic allergic alveolitis.^108,111,113,118^ In addition to the accumulation in the lungs, MP particles are swallowed by macrophages and, since particles of 15–20 microns in size are toxic to them, MP end up being transported also in the lymphatic and cardiovascular system.^119^ More recent findings can be found in Yang *et al.* (2022).^120^ It must be considered that studies on the effects of MP for inhalation require further investigation in order to quantitatively assess the exposure doses and fully understand the real risks for human health.

#### Ingestion

As mentioned above, MP can be easily ingested by a large number of organisms due to their very small size, which is considerably smaller than macroplastics. The main sources of microplastics ingestion for humans are food and water.^121^ Cox (2019)^122^ points out that exposure levels differ between sex categories and age groups since different are lifestyles and diets, with a maximum exposure recorded for male and female adult categories. Recently, Koelmans *et al.* (2019)^123^ showed that the amount of MP particles varies in different water matrices, such as treated and untreated wastewater, surface water, tap water and bottled water. Regarding drinking water, a big distinction must be made between tap and bottled water. The number of MP particles, in fact, ranges from 0–61 particles per L for tap water and from 0–10 000 particles per L in the case of bottled water.^111^ Currently, the methods used for the treatment of raw water, known as WTPs (water treatment plants), are able to remove at least 70% of the MP present. This percentage can actually increase further, depending on the method type, from 70% in the case of WTP1 (sand filtration) up to a maximum of 85–86% for combined WTP2 (sedimentation + sand filtration) and WTP3 (flotation + sand filtration) treatments.^124^ Nevertheless, the high number of MP present in bottled water comes mostly from the container itself and to a lesser extent from the water distribution and bottling processes. In support of this, it has been found that about 80% of MP in bottled water consist of PET, PP, PE, the materials of which caps and bottles are made.^125^ Furthermore, the same study^125^ has interestingly shown that a considerable number of MP particles are also present in water bottled in glass, leading to the assumption that the plastic cap is the one releasing the greatest amount of microplastics (50 particles per L).^111^ Turning to the numbers, Cox *et al.* (2019)^122^ showed that on average 90 000 are the MP particles ingested annually by humans only by drinking bottled water, while the amount is about 22 times lower (4000) by drinking tap water.

It is interesting to point out how the presence of MP has also been highlighted in other food beverages such as energy drinks, bottled tea, wine and beer. For example, 2563–5857 MP particles per L were found in white wine from Italy, where the major amount comes from the synthetic stoppers often made of polyethylene, and 10–256 particles per L were found in beer from Germany, even if, in this case, MP sources can be different.^126^

The presence of MP has also been well documented in food. The first evidence dates back in 1960, when plastic fragments were found in the guts of sea birds (the global annual plastic production was less than 25 million tons at that time).^7^ Today, the greatest amounts of MP is found in food products such as crustaceans and commercial fish,^127–129^ bivalves,^130,131^ salt^132^ and sugar.^133^ Among these sources, sea salt and seafood have aroused particular interest and a greater number of studies have been focused on them. An average concentration of about 0–20 000 MP particles per kg was found in salt but these values can easily vary depending on the geographical origin. For example, Kim *et al.* (2018)^134^ showed that sea salt from the coasts of eastern and southern Asia has the highest number of MP particles compared with sea salt from European, North American and Australian coasts. It should be noted that this geographical distribution of MP in sea salt can easily be correlated with the evidence from Lebreton *et al.* (2017), according to which rivers flowing near the coasts of eastern and southern Asia provide the largest contribution to plastic release into the oceans.^64^ Seafood is currently ranked as the third cause of human MP consumption, after bottled water and alcohol.^122^ Rochman *et al.* (2015)^135^ found that each fish species can contain from 25% to 33% of MP, but this percentage rises to about 60% if all marine species are considered. Also, these percentages can change considerably according to the geographical food habits, as some urbanized populations have significantly higher average consumption of seafood than others. For example, anchovy, a fish typically eaten without removing the digestive tract, is a common food in Japan. Given that 2 MP particles can be found in each anchovy,^136^ it was estimated that Japanese seafood consumption can result in the accumulation of 154 MP particles per day.^122^

In general, about 90% of ingested MP are eliminated from the human excretory system *via* the feces. The removed MP are usually larger than 150 μm in size while smaller MP can be absorbed much more easily by the human body. In fact it has been shown that MP particles with dimensions smaller than 150 μm can easily cross the gastrointestinal epithelium, MP particles with dimensions of the order of 10 μm can pass through the placenta and the blood–brain barrier and MP particles of smaller dimensions at 2.5 μm are able to reach the systemic circulation by endocytosis.^111^ Absorption of MP by the intestinal mucosa is certainly the main route through which MP remain within the human body. In addition, paracellular transport in the intestine and cellular uptake in the lungs must be taken into consideration. Paracellular transport in the gut, in the form of persorption, occurs when the MP particles are of the order of 130 μm.^122^ For dimensions of about 10 μm or more, the main route of entry is the absorption by specialized M cells of intestinal lymphoid tissue (Peyer's spots of the ileum) while smaller MP particles (few microns) can be directly taken up by the gut and lungs cells.^108,122^ These compounds are particularly harmful and can cause various types of physiological damage as listed below^108,122^

Disruption of immune function: prolonged exposure to MP can lead to autoimmune diseases or immunosuppression,^109,113^ the causes of which are attributable to the release of immunomodulators, to the erroneous activation of immune cells and to oxidative stress.^137^ Autoimmune rheumatic disease^138^ and systemic lupus erythematosus^139^ can also occur.

Translocation to distant tissues: translocation occurs through the circulatory system and produces inflammation following which an immediate increase in the permeability of cell membranes is recorded.^109^ Studies have shown that the presence of MP in the circulatory system can cause occlusions and vascular inflammation,^140^ pulmonary hypertension,^117^ systemic inflammatory response and blood cell cytotoxicity through internalization.^141^ Prata *et al.* (2020)^109^ also demonstrated that translocation can lead to chronic inflammation, reduced organ function and increased cancer incidence. Furthermore, the presence of MP in bone can cause bone loss by activating osteoclasts.^142,143^

Altering metabolism and energy balance: exposure to MP can cause an alteration of human metabolism either directly or indirectly. In the first case, the activity of metabolic enzymes is compromised or in any case modified, while in the second case the energy balance, the homeostatic balance between energy produced and energy consumed, is interrupted. It has been shown that the most frequently occurring effects are a lowering of nutrient intake, an alteration of the activity of metabolic enzymes and an increase/decrease in energy consumption.^108^

Oxidative stress and cytotoxicity: oxidative stress and, consequently, cytotoxicity are the most common effects generated by MP exposure. Oxidative stress occurs as MP particles can release ROS (reactive oxygen species), created by the manufacturing process of plastics, weathering or exposure to UV light,^144^ oxidizing chemicals (such as metals), which easily adsorb on their surface, or oxygen-containing radicals, produced as a result of the inflammatory response.^145,146^ Sternschuss *et al.* (2012)^147^ showed that the inflammatory response of the human body due to the fitting of limb and joint prostheses containing MP led to the release of free radicals and acute toxicants which completely degraded the prosthesis itself. Oxidative stress and inflammation can lead to cytotoxicity. MP particles, in fact, can be digested and internalized by macrophages.

Neurotoxicity: neurotoxicity occurs due to chronic MP exposure. Direct contact with translocated particles or alteration of the levels of proinflammatory cytokines can lead to the activation of the immune cells in the brain and to oxidative stress, with consequent permanent damage at the neuronal level.^108^

Reproductive toxicity: Chang *et al.* (2020)^148^ have shown that MP particles can accumulate in the gonads, leading to a reduction in their reproductive capacity due to the alteration of energy metabolism and oxidative stress.

Carcinogenicity: Chang (2010)^149^ showed that chronic irritation and oxidative stress generated by the presence of MP in the human body can lead to the release of pro-inflammatory mediators. Such mediators can involve angiogenesis which, in turn, can lead to the formation of tumors. In 2018, Prata^113^ then demonstrated that the same effects, *i.e.* chronic irritation and oxidative stress, can cause the onset of tumors as a result of DNA damage.

Indirect effects through acting as vectors of toxic chemicals and microorganisms: as mentioned above, the MP particles can contain additives, chemical pollutants, metals or other substances that are highly toxic to humans. From a purely numerical point of view, the exposure to toxic agents from MP is irrelevant when compared with the daily intake of MP for food or dust. However, it is the prolonged exposure to toxic agents that can cause the numbers to grow to such an extent that they are considered a real risk factor.^150^ It has been shown that BPA, BPS and in general many plastic additives are endocrine-disrupting chemicals (EDCs), leading to a higher incidence of early onset of puberty and genital defects, blood infection and breast cancer.^151,152^ Not only toxic agents: the large surface area of MP can also constitute fertile ground for numerous microorganisms such as vibrio spp., one of the most virulent bacteria,^153^ or *Folsomia candida*, a soil organism which acts as a promoter of gut microbiome's activities.^154^

#### Dermal contact

Absorption of MP through the skin is not possible: it has in fact been shown that only particles smaller than 100 nm are able to cross the dermal barrier.^155^ However, personal care products such as facemasks, facewashes, hand cleansers and toothpaste contain MP, which can cause skin damage due to local inflammation and cytotoxicity.^72^ Schirinzi *et al.* (2017)^156^ showed that oxidative stress can also occur in the cells of the dermal epithelium. It should be noted that even plastic products used in surgery can often lead to severe inflammation. To date, there are few studies on the risks due to exposure of MP for dermal contact and therefore greater efforts are required in the coming years to have a much more satisfactory picture of the situation.

## Impact on marine habitat-forming species

In the last two decades, numerous studies have been carried out to monitor the presence of plastic in the oceans and the damage that its presence causes to the marine biota. What emerges very clearly is that there is no place that plastic has not reached.^75,80,157–163^

The greater the amount of MP in the marine environment, the greater the bioavailability for marine biota. Numerous studies based on the analysis of the stomachs of marine organisms have revealed the presence of MP inside them, confirming that marine organisms can ingest them.^98,164–166^ This is attributed to the fact that marine organisms mistake microplastic particles for food due to their small size, and thus ingest them. The MP uptake by marine biota depends on numerous factors such as density, shape, color, charge, abundance and aggregation of microplastic particles.^167^ Regarding the density, it has been shown that usually the lower density particles are ingested by copepods and are subsequently excreted in the faeces. The sinking speed of these fecal pellets is strictly dependent on the density of the microplastics inside them and this means that they can in turn become nourishment for copepods, polychaetes, crustaceans but also for fish. High density microplastics are usually ingested by benthic invertebrates and deep ocean biota. MP shape influences their dynamics indirectly as it is responsible for the bioavailability and distribution of the MP particles in the marine environment. Generally, spherical shaped particles tend to sink much faster than thin films and plastic fibers of equal density. Shape is also related to the time MP persist in the marine organisms after ingestion and to the excretion process. For example, the amphipod *Hyalella azteca* is able to ingest both MP fibers and spheres.^168^ However, the clearance time in the case of fibers is much longer than that for the spheres, thus implying, due to their difficulty in being excreted, fibers have greater toxicity. Generally, irregularly shaped MP particles are more toxic than those of regular shape due to the greater difficulty that organisms have in the process of egestion. MP color is one of the most striking demonstrations of how ingestion of microplastics by marine organisms is due to the fact that they often mistake MP for food. Darker colored MP, especially green microplastic fibers, are easily ingested by flathead gray mullet (*Mugil cephalus*) as they closely resemble marine plankton, which this animal normally feeds on.^169^ Another example is that blue polyethylene fragments are ingested by about 80% of the Amberstripe scads (*Decapterus muroadsi*) who mistake them for blue copepods, their prey, being similar to them in color and size.^170^ Numerous studies have been carried out and are currently underway to understand how harmful are the side effects on marine biota due to MP ingestion.^72,167,171^ It has now been widely demonstrated that damage to the organism can be both physical and chemical and can lead, in the most serious cases, to its death.

Once ingested, the fate of microplastics can follow several paths as described below:

(a) At best, they can be eliminated from the marine organism by excretion or production of pseudofaeces, thus not leading to significant side effects except for the alteration of the organism's energy flow. The function of excretion, in fact, performs two main tasks: to separate the organic material from the inorganic components, that is the MP, and to act as a cleaning mechanism preventing an accumulation of particulate material at the level of the organism gills. It is often observed that excretion occurs a few hours after ingestion of the MP particles. This is due to the fact that, most likely, such organisms are able to recognize MP as low-energy food and consequently remove them.^151^ However, pseudofaeces, loaded with microplastics, constitute in turn food for other marine organisms and the problem therefore moves to higher trophic levels.

(b) MP are retained by the organism and cause toxic effects. What occurs is the accumulation of MP in the digestive system, producing physical damage such as injury and clogging, and the appearance of adverse effects such as pathological and oxidative stress, inflammation and liver toxicity, reduced growth rate, false satiation, blocked enzyme production and reproductive complications.

(c) After the digestion process, previously retained MP enter the bloodstream and reach different internal organs and tissues through the translocation phenomenon.

(d) According to the food chain, marine organisms that have ingested MP can in turn constitute food for other organisms, which therefore ingest MP indirectly.

As already pointed out, the toxicity of MP is also attributable to the contaminants and pollutants that they are able to retain on their surface and that can be released into the organism once MP are ingested.

The entire marine biota is affected by the presence of MP. In fact, it has been shown that MP can be ingested by a large number of invertebrate and vertebrate marine organisms including coral, zooplankton, shrimps, bivalves, copepods, mussels, lugworms, oysters, fishes and by larger animals such as turtles, manatees, otters, snakes, sea lions, penguins, seals and whales.^1,160,172^

Currently, with an extension of about 0.2% over the entire oceanic area, coral reefs, having the task of mitigating the effects of the ocean destructive forces on the coasts, constitute the natural habitat of about one third of all marine fish species and thousands of other marine organisms. They mainly feed on phytoplankton and zooplankton, copepods and amphipods. These organisms are all capable of ingesting MP which, consequently, end up in corals too. MP accumulate in the digestive tract of corals, leading some species of hard corals to death and consequently reducing the biodiversity of these environments.^173^

Among the marine organisms, the most affected by MP contamination are planktons, a worrying fact especially considering that they are the nourishment of a large part of the marine biota. Law (2010) showed that microplastics were found in about 60% of the plankton living between the North Atlantic Ocean and the Caribbean Sea.^81^ In the report of the Norwegian Institute for Water Research of 2014, Nerland *et al.* demonstrated that MP can cross both cell membranes and cell walls and interfere with the photosynthesis process by reducing the concentration of chlorophyll in the green alga *Scenedesmus obliquus*.^174^ Adverse effects include disturbed feeding and digestion due to the accumulation of MP in the digestive tract or in the gut of zooplanktons.^72^ Zooplanktons expel MP in the form of faecal pellets. This would not be a problem if it wasn't for the fact that these pellets are nourishment for benthic and pelagic marine organisms, which therefore ingest MP indirectly.^175^

The group of benthic organisms is characterized by a remarkable biodiversity, covering about 98% of the entire marine biota, and includes invertebrates such as lobsters, blue mussels, oysters and barnacles. They are all capable of ingesting MP.^176,177^ A 2014 study found that farmed blue mussels from Germany, near the North Sea, contain a number of MP particles equal to 0.36 ± 0.07 particles per g (wet weight) while it is equal to 0.47 ± 0.16 particles per g (wet weight) for oysters farmed in Brittany and France near the North Atlantic Ocean.^178^

MP were found in approximately 36.5% of pelagic and demersal fishes^179^ and in approximately 18% of top predators in the central Mediterranean Sea, such as bluefin tuna (*Thunnus thymus*), swordfish (*Xiphias gladius*) and albacore (*Thunnus alalunga*).^180^ It has been observed that MP can accumulate in the gut of fishes, causing starvation and malnourishment which can also lead to their death.^181^

Sea birds, which feed on what they find on sea surface, are also affected by the presence of MP in their digestive tract.^72^ 30–35% of the MP particles are in the form of industrial pellets. In addition to the process of excretion through the production of faeces, sea birds tend to remove MP from the digestive tract by regurgitation.^182^

At this point the presence of MP was also ascertained in marine mammals, such as whales, harbor seals, sea turtles, and polar bears.^1,183–186^ They can ingest MP through three different phenomena: inhalation, feeding or trophic transfer from prey.^187^ In 2014, Nerland found that around 60.5% of turtles in Brazil contain MP in their digestive tracts.^174^ Whales are another marine mammal particularly affected by MP contamination. This is due to the fact that they often perform a filtering function for seawater so they tend to ingest MP, and to accumulate them in their stomach and liver.^188^ Due to the high ingestible quantities of MP, cetaceans are often used as probes to check the level of plastic pollution in the areas where they live by measuring the quantity of phthalates in the blubber of stranded fin whales (*Balaenoptera physalus*).^188^ However, these data currently need to be considered cautiously as phthalates can come from various sources.

## Removal and treatment technologies

The removal of MP from water is a key challenge in the struggle to mitigate environmental pollution. Traditional water treatment plants are not designed to remove MP and they have been globally identified as a major source of MP to the environment.^16,124,189,190^ Hence, it is important to understand our current ability to remove these pollutants *via* engineered treatment methods and identify key opportunities to reduce their concentration in receiving water bodies. For this purpose, several physical, chemical and biological processes for the treatment of MP contaminated water are reviewed below.

### Physical processes

Physical separation processes for primary wastewater treatment include screening, skimming and sedimentation. Using these methods, fast and low cost filtering of large contaminants is possible. The ability to remove MP depends on the wastewater characteristics as well as the type of treatment process applied.

#### Screening and settling

Traditional screening and settling methods may remove significant portions of suspended solids in water. For example, Liu *et al.* (2018)^191^ observed that the average size and number of MP fragments present in the water in a Chinese WWTP decreased by 58.8% and 40.7%, respectively, after initial screening and sedimentation. The particles removed were mainly large but the authors also observed the adherence of MP to suspended solids in the water, allowing physical removal of smaller fragments and fibres that might not be expected otherwise. Moreover, screening and sedimentation have reportedly removed up to 88% of anthropogenic solids in a treatment facility in the United States (although this classification includes more than just MP).^192^ The importance of plastic density as related to settling velocity during the sedimentation step is highlighted by Iyare *et al.* (2020).^193^ The huge variety of polymers in terms of density, shape and type that are found in wastewater can substantially influence their physical separation from water. Settling alone removes an average of 72% of MP particles present in sewage influent but the smallest particles (<27 μm in diameter) are likely to pass unimpeded. As well as failing to capture the smallest particles, reliance on traditional separation processes to remove MP in this way presents another problem of transferring the pollutants from liquid to a concentrated solid waste.^194,195^ This can become a serious environmental problem when sludge is recycled for land treatment or disposed of inappropriately; thus potentially allowing MP to find their way back into the water system.^196^

The difference in density between MP and the natural organic matter of water presents an opportunity for physical removal. Several lab scale studies have applied enhanced density separation in salt solution as an isolation method for MP samples in complex matrices such as wastewater or natural water.^197–199^ Scaling up this method is challenging due to the large volumes of high cost salt solutions required.^200^ Alternatively, enhanced settling *via* coagulation seems promising for large-scale application. Using traditional Fe salt coagulation conditions, the removal of polyethylene (PE) MP was found to be poor with less than 15% removal.^201^ However, in the same experimental setup, using a high dose anionic PAM coagulant, removal was increased to over 90%. This can be attributed to the low density of PE that inhibits settling of the produced flocs and reduces the efficiency of the coagulation treatment. An impact of this effect was also observed by Ma *et al.* (2019),^202^ where the highest efficiency removal of approximately 60% was achieved for the smallest sized MP. In addition to density, the shape of MP is likely to affect greatly the settling behaviour. Skaf *et al.* (2020) investigated the removal of both fibres and microspheres of PE using an alum coagulation method and they found that both could be adequately removed by sweep flocculation.^203^ Less traditional coagulation-like methods such as the bioinspired agglomeration process developed by Herbort *et al.* (2017, 2018)^204,205^ suggest targeting specific MP in water. Electrocoagulation methods have also been explored and over 90% removal of PE beads was reported.^206^ Despite the progress in the area, the main problem with coagulation based methods is the highly variable nature of MP surfaces. The surface charge is not easily predicted meaning that coagulants specifically designed for MP removal are less efficient than for the removal of organic material.

#### Filtration and adsorption

Some developed materials for adsorption and activated filtration aim to target MP in water. Biochar offers a low cost option for removal of many pollutant classes, including MP.^207,208^ Sun *et al.* (2020)^209^ engineered an adsorption separation process relying on a biodegradable chitin and graphene oxide sponge which is capable of removing up to 89% polystyrene from water. Batch filtration was proposed using a Zr-MOF system with over 95% efficiency for removal of PVDF microplastics.^210^ While promising in performance, adsorption and batch filtration treatment pose a challenge in scaling up to meet the needs of a full scale WWTP.

Membrane separation of MP is an option already employed at scale. Malankowska *et al.* (2021) review in detail the advances made in microfiltration, ultrafiltration and nano-filtration methods in the removal of MP.^23^ Michielssen *et al.* (2016) suggest retrofitting WWTPs with granular sand filtration and membrane filtration has the highest potential for MP removal in a meta-analysis of available technology.^192^ This finding was backed up for large scale studies in a comparison of municipal WWTPs where rapid sand filtration removed 97% of MP.^16^ However, the high potential removal efficiency comes at the cost of membrane pore blockage and resulting flux reduction when applying high-pressure filtration techniques. Such issues arose within just 48 h of ultrafiltration when 38% flux reduction occurred due to MP blockage of pores during treatment of MP contaminated water.^211^ MP interaction with organic matter in the water may also enhance the rate of membrane fouling. The size of MP in the raw water were found to be the major factor influencing fouling during freshwater ultrafiltration treatment with the most severe effects at a MP size of 1 μm.^212^ However, continuous filter module rotation could significantly reduce the fouling and the treatment became more attractive for large scale application. MP in landfill leachate were successfully removed using membrane separation. Interestingly, the researchers identified the re-release of captured MP from the filter back into the effluent suggesting that the particles are not permanently immobilised onto the membrane and may still be released into the environment. Similar removal and re-release behaviour occurred in ultrafiltration membrane treatment of drinking water as well as some MP penetrating the membrane itself.^213^ Despite a 98% removal by reverse osmosis treatment, fibers of plastic under 200 μm passed unimpeded indicating that membrane processes alone cannot completely remove MP.

### Chemical processes

Effective chemical degradation of polymers could mineralise MP during water treatment and avoid transferring any waste to a new solid phase. Advanced oxidation processes (AOPs) are an effective means to remove biologically recalcitrant contaminants by the generation of non-selective, highly reactive radicals. AOPs are effective in the removal of emerging organic pollutants, such as antibiotics, personal care products, trace organics.^214–216^ Recently, their potential application in the rapid degradation of MP is drawing attention.^217^ During chemical degradation, the surface of the polymer undergoes chemical change and the MP subsequently fragment.

#### Photocatalytic treatment

The majority of research published in AOP treatment of MP focuses on photocatalytic methods and is summarised in Table 2. Titania (TiO_2_) based photocatalysis is the most common treatment due to its low cost and relatively high radical generating ability.^218^ Due to the need for a surface contact during heterogeneous photocatalysis, the morphology of the catalyst is an important area of focus with researchers exploring the use of nanotubes and nanoparticles.

**Table 2:** Advanced oxidation treatment methods for microplastic removal from wastewater

AOP	MP targeted	Monitoring methods	AOP performance	Other parameters	Ref.
C,N–TiO_2_ photocatalysis	Polyethylene (PE) beads	Mass loss, FTIR measured carbonyl index (CI), microscopy	50 h, mass loss 72% and large increase in CI	Visible light LED (400–800 nm), temperature 0–40 °C, pH 3–11, lamp distance 25 cm, 4 L^−1^ MPs, 4 g L^−1^ catalyst (optimum removal at pH 3, 0 °C)	219
N–TiO_2_ photocatalysis	PE beads and flakes	Mass loss, CI	50 h, mass loss < 5% for all (4.6% HDPE and 1.8% LDPE)	Visible light LED (400–800 nm), pH 3, room temperature, 4 g L^−1^ MPs, lamp distance 21.5 cm	220
ZnO nanorod photocatalysis	PE film	Microscopy (SEM), mechanical change, CI	175 h, CI increase of 30%	Visible light 50 W dichroic halogen ambient air. Deionised water	224
N–TiO_2_ photocatalysis	PE beads	Mass loss, SEM, CI	8 h, mass loss < 3% in aqueous solution	27 W visible fluorescent lamp (400–800 nm), room temperature, lamp distance 12 cm, 2 g L^−1^ MPs	223
PMS/carbon nanospring photocatalysis	PE	Mass loss, CI, SEM	8 h, 40% mass loss	pH 3–11, MPs 5–12 g L^−1^, temperature 25–160 °C (>150 °C optimum)	227
ZnO photocatalyst supported on glass fibre	Polypropylene (PP)	Size change, CI, SEM	456 h, 65% reduction in volume	Visible light 60 mW cm^2^, 300 mL min^−1^ continuous flow treatment of 10^4^ particles per L	225
TiO_2_ nanoparticle photocatalyst film	Polystyrene (PS)	Diameter change, DRIFTS, GC-MS	24 h required for removal of 400 nm starting diameter particles	254 nm UV irradiation	221
ZnO nanorod photocatalysis with Pt modification	PE film	CI, SEM	175 h	Visible light 50 W, room temperature, lamp distance 10 cm	222
TiO_2_	Polyamide (PA) fibre	Mass loss, CI, total organic carbon (TOC), SEM	48 h, 94% mass loss	UVA irradiation, room temperature, 5 lamp photo reactor	226
Modified TiO_2_ photocatalysis	Poly(methyl methacrylate) (PMMA), PS	TOC	7 h, flow reactor	UVA irradiation 112 W m^2^	229
Hydroxy-rich ultrathin BiOCl photocatalysis	PE	Mass loss	5 h, 6% mass loss	250 W of 420 nm irradiation; MP and catalyst 1 g L^−1^	228
Photo-Fenton	PS	SEM, CI, HPLC/MS, contact angle, C : O atomic ratio (XPS)	108 h, CI increase	500 W mercury lamp, 12 g L^−1^ MPs	231
Fenton	PS and high density PE	Size distribution, CI, C : O ratio by XPS	1–30 days	pH 4, 3 mM Fe, 4.5 mg mL^−1^ H_2_O_2_	232
Fenton	PE, PP, polyvinyl chloride (PVC), nylon	Microscopy	10 min, minor surface area decrease	Room temperature, pH 5, 7 g L^−1^ MPs, 3–10 mg mL^−1^ Fe	233
Thermal Fenton	PE, PS, PP and polyethylene terephthalate (PET)	Mass loss, CI, DSC to determine crystallinity, XRD, SEM, Raman spectroscopy, particle sizing by zetasizer	16 h, 96% mass loss	4 mM Fe^2+^, 300 mM H_2_O_2_	234
Heterogeneous photo Fenton/photocatalysis	PP, PVC	FTIR, particle diameter by microscopy	7 days, 94–96% size reduction	Nano zero valent iron and combined with ZnO/SnO_*x*_ photocatalysis, 60 mW cm^2^ visible light	235
Ozonation	PE	FTIR, XPS	60–180 min, increase in CI	Ozone 4–7 mg L^−1^	236
Ozonation	Mixed MPs obtained from real wastewater	Particle counting	99.2% removal after tertiary treatment (this includes removal *via* other methods during the treatment process)	Ozone 12.6 mg L^−1^ for 1 min during tertiary treatment	237
Ozonation, H_2_O_2_/ozone	PE, PP, PS	Adsorption, XRD, SEM, FTIR	10 min, ozone dose of 88 mg L^−1^	O_3_ : H_2_O_2_ molar ratio of 0.5	238

Using TiO_2_ doped with C and N, a significant loss of mass of 72% was lost from high density PE beads after an extended treatment time of 50 h.^219^ The authors enhanced performance by lowering the water temperature and pH which was attributed to the enhanced radical forming ability of the optimised process as well as changes to plastic properties at low temperature. Nitrogen doped TiO_2_ was also utilised in a study focusing on the effect of plastic shape on removal rate.^220^ By considering the surface area difference between samples (beads *vs.* flakes) as well as the MP particle size, it was concluded that light illumination was the main factor affecting removal. Furthermore, it was observed that the low density PE flakes, an insoluble water pollutant, were floating on the water surface blocking light and oxygen from reaching the submerged catalyst. This probably resulted in generating fewer radicals thus yielding a removal efficiency less than 5%. Similarly, a low removal in water was also observed during treatment of polystyrene (PS) with TiO_2_, which was attributed to low transmittance of UV light.^221^ Removal of the MP from water by physical means prior to AOP treatment allowed effective oxidation but this can limit the practical applicability of the method at large scale.

Other catalyst doping strategies have been explored such as the use of Pt doped ZnO based AOP.^222^ It was found that the proportion of carbonyl and vinyl surface groups on a low density PE film was increased when treated, which indicates surface oxidation. As well as doping, the effect of synthesis conditions on MP removal has been explored. The synthesis method employed for catalyst preparation was investigated by comparing traditional sol–gel and bio derived N-doped TiO_2_, with an improvement in MP removal observed for the latter green-synthesised material.^223^ Synthesis of ZnO catalysts was tuned to produce a variety of nanorod lengths and the effect of their morphology on plastic removal was studied.^224^ High surface area ZnO supported on a glass fibre substrate enabled trapping of low density MP in place in contact with the catalyst for continuous flow treatment.^225^ In the first AOP treatment specifically reported for fibre degradation, a combination of UVC irradiation and TiO_2_ photocatalysis achieved 97% mass reduction of polyamide in 48 hours.^226^ Less common materials have also been investigated such as a PMS carbon nanospring based catalyst and hydroxy-rich ultrathin sheets^227,228^ and TiO_2_ supported on β-SiC alveolar foams.^229^

#### Fenton and Fenton-like treatment

Fenton based AOPs combine hydrogen peroxide and Fe(ii) ions to produce oxidizing hydroxyl radicals, activated by light or heat.^230^ A few of these treatment methods have recently been applied for MP removal and are summarised in Table 2.

Many of the Fenton based treatment processes reported in the literature were developed with the aim of accelerating the ageing of MP in order to study the change in adsorption or transport behaviour.^231–233^ Although not aimed at removing MP completely, these studies provide important insight into how plastic degradation is initiated by Fenton processes and could be harnessed for water treatment applications. For example, during Fenton treatment of PS and HDPE over long timescales, issues with the deposition of ferric hydroxide were observed thus leading to poor removal from water, despite steps taken to mitigate this.^232^ Thermal methods of activating the Fenton reaction can offer an alternative mechanism of action, as explored by Hu *et al.* (2022).^234^ A high mass loss of plastic was observed for a range of polymers but the treatment required the use of high temperatures in order to break down the crystallinity of the plastics, which limits its applicability at large scale. Furthermore, combining heterogeneous systems (*i.e.* solid iron on a supporting matrix) with photocatalysis resulted in significant reduction in size of PVC and PP particles after 7 days.^235^

#### Ozone and peroxide based treatment

Ozone has been applied either on its own,^236,237^ or in combination with radical generating hydrogen peroxide^238^ to degrade MP. In the latter case, a 10 minutes treatment affected the surface chemistry and associated adsorption behaviour of several types of plastic. Although the treatment is not directly designed for removal, the resulting change in adsorbance properties is relevant to understanding how MP treated with AOPs may carry additional micro pollutants through the water treatment process and therefore extending the treatment duration may be an option for investigation. Similarly, Zafar *et al.* (2021) considered the effect of ozone treatment on the surface of PE particles and found that reaction time was more effective in increasing the oxygen prevalence on the surface compared to ozone dose.^236^ Hidayaturrahman *et al.* (2019) reported that ozonation combined with primary, secondary and coagulation steps removed 99.2% of MP in a full-scale treatment plant.^237^ While reasonably high removal performance is possible, slow reaction rates currently limit the applicability of AOPs in water treatment. Long contact times of several hours are not feasible in a high throughput system. In order to bring AOPs into the focus for water treatment, improvements to the rate of degradation are essential.

### Biological processes

Biodegradation of plastic is an intensive area of research and has potential for application in wastewater treatment.^239–241^ Traditional water treatment systems typically involve an element of biodegradation for the removal of organic matter. In most cases, these systems fail to adequately remove MP, leading researchers to explore alternatives. In a systematic review for MP removal, Iyare *et al.* (2020)^242^ identified that 19 out of 21 traditional wastewater treatment systems included activated sludge treatment as a secondary step. On average, activated sludge could reportedly remove 16% of MP from the water (across a broad range of 0.2–52%). Alongside limited effectiveness, relying only on traditional water treatment methods like this can create further challenges. Physical transfer of MP out of the wastewater moves the plastic pollution into another part of the water treatment process – the sludge. Also, MP can affect the activity of bacteria used for organic matter decomposition as well as cause issues downstream in sludge treatment.^191,243,244^ Wei *et al.* (2021) showed that PET MP inhibited aerobic digestion of waste activated sludge (WAS) by approximately 10%, which was attributed to its influence on microbial communities.^244^ In another study, anaerobic digestion of WAS was found to be impeded by the presence of PVC MP. Moreover, bisphenol A leaching out of the plastics was linked to a decrease in methane production and inhibition of the treatment in this case.^245^ Biofiltration of wastewater was identified as a more effective biological secondary step for MP removal than activated sludge with an average of 19% from the treatment systems surveyed.^242^ However, the presence of MP in these systems drastically impedes the effectiveness of treatment for other contaminants. Membrane bioreactors (MBR) reportedly experience immediate decline in removal of organic matter from 80% to below 50% upon addition of PVC MP.^246^ This was attributed to an increase in membrane fouling as a result of MP build up. In order to avoid these knock on effects to treatment system performance, the presence of MP needs to be taken into account when (re)designing water and wastewater treatment processes. Developing biological treatments specifically aimed at combatting plastics is a challenging area of research but is clearly required to overcome the shortcomings of traditional WWTPs. Outwith the context of wastewater treatment, bacteria, bacterial consortia^247–250^ and fungi^251,252^ have been investigated for their ability to degrade plastics. Microbial digestion is an extremely attractive option to solve the problem of plastic waste in a potentially sustainable way. One of the main hurdles in each case is the lengthy treatment times required (many reported biodegradation options take weeks or months) for it to be effective for large scale application. In 60 days, PE was biodegraded by a specifically isolated microbial consortium to reduce its mass by just 14.7%.^250^ PET increased crystallinity and reduced in particle diameter as a result of bacterial degradation in a high pH process over 48 h but complete removal was not achieved.^253^ Using a surfactant to improve interfacial activity, bacterial degradation of PET reached 11% mass loss in 5 days.^254^ As well as the lengthy digestion times required, biological systems are likely to struggle to cope with the diverse nature of our plastic waste. Enzymatic digestion relies on specific target groups in the polymer chain being broken. To tackle the huge range of polymers present in our waste, a great many different plastic digesting microorganisms will be required.^255^ As it stands, these engineered systems are not currently capable of treating the high flow of microplastics in our water treatment systems due to the long timescales required but are under constant development as reviewed comprehensively elsewhere.^256^

**Fig. 7:**
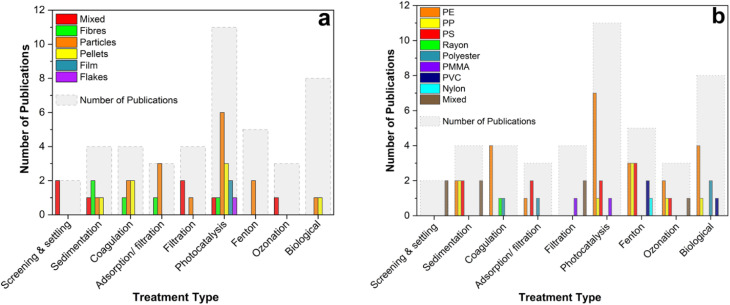
(a) Publications reviewed considering water treatment for the removal of microplastics broken down by plastic shape. (b) Publications reviewed considering water treatment for the removal of microplastics broken down by polymer type.

**Fig. 8:**
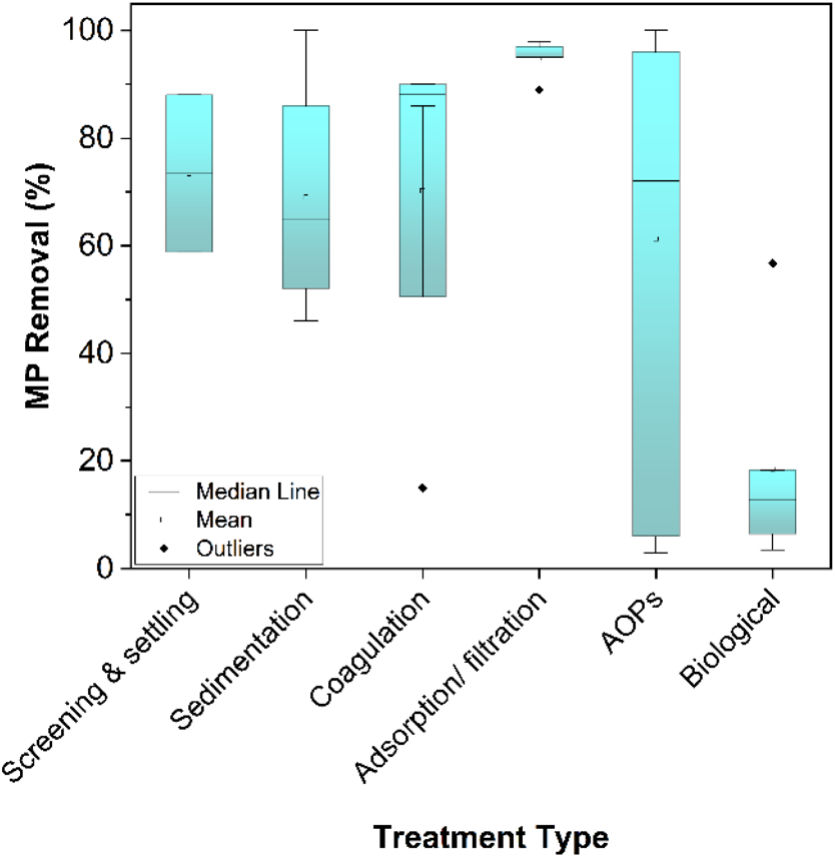
Summary of the reported microplastic removal performance of water treatment technologies reviewed.

As previously discussed, a broad range of polymer types and MP shapes are present in the environment. Lab scale treatment studies have focused on a number of different MP subtypes. It is important to consider the effect of this when evaluating the efficacy of a reported treatment system. Fig. 7 shows the papers discussed in this section, broken down by which MP types and shapes are focused on. Furthermore, Fig. 8 highlights the performance of treatment technologies reviewed here in terms of MP removal from water. It can be observed that primary treatment (screening, settling, sedimentation) can remove about 50–90% of MP from wastewater, while adsorption and filtration are able to remove more than 95%. The percentages for advanced oxidation processes can span from very low to almost complete removal of MP. For biological engineered technologies the removal can be up to 20%. Physical treatment methods offer reasonably consistent high removal rates but are hampered by the production of solid waste and process problems such as membrane blockage. Chemical and biological treatment methods are an area of intense research and have the potential to effectively destroy MP if the rate of degradation can be significantly improved. Further research into these important water treatment systems is required to further understand their potential in tackling MP pollution.

### Monitoring and treatment technologies: future perspectives

Currently, the most common chemical techniques used for the identification of MP are generally based on electronic microscopies (SEM, TEM), on Fourier transformed infrared (FT-IR) and Raman spectroscopies (micro-Raman and micro-FT-IR for smaller particles) and on thermal identification (DSC, TGA). Detection of polymer type and better quantification of MP in collected samples are achieved by using more performant analytical techniques, such as thermal chromatography, usually coupled with mass spectrometry (Pyro-GC/MS), to ensure highly sensitive MP detection.^257–261^

Considering the global emergency due to plastic pollution, it is increasingly important for the scientific community to develop and/or improve technologies and methods that can guarantee a better quantification of MP, both during the extraction and analysis phases.^262^

In this regard, Silva *et al.* (2020) implemented the μ-FTIR hyperspectral imagining technique using a new machine learning approach to quantify and characterize microplastic particles.^263^ Cashman *et al.* (2022) developed a new method of extracting MP from marine sediments in order to obtain significantly higher extraction yields than standard methods.^264^ Castelvetro *et al.* (2021) proposed an alternative approach combining wet chemical techniques of extraction with analytical quantification techniques such as reverse phase HPLC and size exclusion chromatography for the determination and quantification of MP in marine sediments and freshwater.^261^

At the same time, the world scientific community is also striving to develop new technologies focused on preventing plastic waste from entering the environment. The latest scientific advances and technologies available in this area are collected in the Plastic Pollution Prevention and Collection Technology Inventory (https://nicholasinstitute.duke.edu/plastics-technology-inventory), created to help local governments and non-governmental organizations with the aim of helping to solve the hotspots of marine plastic pollution.^265^

The Plastic Pollution Prevention and Collection Technology Inventory currently contains 52 technologies (developed starting from 20 July 2020) with the aim of:

(1) Prevent plastic pollution from entering the environment;

(2) Collect existing marine plastic pollution.

The technologies in the inventory are classified according to the remediation strategy (prevention or collection), the plastic type or the inventory category (laundry balls, textile fibers, personal care products, disposable products, *etc.*).

In order to minimize plastic in the environment, the scientific community is also thinking about a final destination for plastics collected through the exploration of innovative recycling solutions, such as plastic-to-fuel and bioremediation.^266–268^

## Conclusions

While progress has been made in addressing the global plastics challenge, commitments from governments and industry will reduce the annual volume of plastic flowing into the ocean by only about 7% by 2040.

A great deal of attention has been paid to tackling the problem of microplastic pollution in water with physical, chemical and biological treatment options explored by recent research. Considering the complexity and scale of microplastics present in our water, a universally effective treatment solution has not emerged. Incorporation of several innovative steps such as advanced oxidation and targeted biological degradation into broader treatment systems may effectively remove microplastics. Updates to traditional methods to deal with an increased plastic load in our wastewater are required or treatment infrastructure faces the possibility of process problems as well as release to the environment.

Together with innovative techniques, existing plastic waste recycling systems should be improved to prevent microplastics release from the inland to the sea and this task can and must be reached in a short time. The same goes for municipal sewage treatment processes which in many cases do not even include microplastics removal units. This environmental problem, common to the governments and the communities of all countries, must trigger practical policies and measures to ensure conservation actions for the aquatic ecosystem.

## Author contributions

Project conceptualization, methodology, and supervision, A. T., M. B., F. C., E. C. and G. D. F.; administration, funding acquisition, E. C. and G. D. F.; methodology, investigation, data analysis, writing—original draft preparation, A. T., M. B., F. C., T. E., E. C. and M. D. B.; writing—review and editing, A. T., M. B., F. C., T. E., E. C., and G. D. F. All authors contributed to the discussion, reviews and approval of the manuscript for publication.

## Conflicts of interest

There are no conflicts to declare.

## Supplementary Material
